# Cowpox Virus Transmission from Rats to Monkeys, the Netherlands

**DOI:** 10.3201/eid1206.051513

**Published:** 2006-06

**Authors:** Byron E.E. Martina, Gerard van Doornum, Gerry M. Dorrestein, Hubert G.M. Niesters, Koert J. Stittelaar, Marno A.B.I. Wolters, Hester G.H. van Bolhuis, Albert D.M.E. Osterhaus

**Affiliations:** * Erasmus Medical Center Rotterdam, Rotterdam, the Netherlands;; † Utrecht University, Utrecht, the Netherlands;; ‡ AAP Sanctuary for Exotic Animals, Almere, the Netherlands

**Keywords:** Poxvirus, zoonosis, nonhuman primates, transmission, rodents, dispatch

## Abstract

We report an outbreak of cowpox virus among monkeys at a sanctuary for exotic animals. Serologic analysis and polymerase chain reaction were performed on blood and swab samples from different rodent species trapped at the sanctuary during the outbreak. Sequence comparison and serologic results showed that brown rats (*Rattus norvegicus*) transmitted the virus to monkeys.

Cowpox virus (CPXV) is a member of the genus *Orthopoxvirus*, family *Poxviridae*, and is antigenically and genetically related to variola virus, vaccinia virus, and monkeypox virus (MPXV). With the eradication of smallpox, routine vaccination with vaccinia virus ceased, which created a niche for animal poxviruses to infect humans. However, cowpox is a rare zoonosis, and infection of immunocompetent persons usually results in localized lesions mainly on fingers, hands, or face. However, in immunocompromised patients, severe generalized infections have been documented (*1**,**2*).

The reservoir hosts of CPXV are wild rodents; cows, domestic cats, and humans are incidental hosts. In Europe, bank voles (*Clethrionomys glareolus*) and wood mice (*Apodemus sylvaticus*) constitute the main reservoirs (*3*), whereas CPXV was sporadically detected in rats (*Rattus norvegicus*) (*4**,**5*). Domestic cats play a role in transmission of CPXV to humans (*6**,**7*). Direct transmission of CPXV from rodents to humans has also been documented (*3**,**5*). In the United States, prairie dogs (*Cynomys ludovicianus*) have been suggested as a potential reservoir for MPXV and are susceptible to CPXV infection by wild rodents (*8*). We report an outbreak of CPXV in nonhuman primates through contact with infected brown rats.

## The Study

In September 2003, three Barbary macaques (*Macaca sylvanus*) at a sanctuary for exotic animals in Almere, the Netherlands, showed multifocal gingival, buccal, and lingual lesions. Typical intranuclear inclusions were found by histologic analysis, and poxlike particles were found by transmission electron microscopy of 6 biopsy specimens from buccal lesions of the same animals. Because of concerns that these macaques were infected with MPXV, additional biopsy specimens of poxlike lesions were obtained for virus isolation and polymerase chain reaction (PCR) studies.

Vero cells were infected with homogenized biopsy samples from the 3 macaques, and cells were monitored daily for appearance of cytopathic changes. Three days after infection, cells showed cytopathic effects characterized by plaques of rounded cells with prominent cytoplasmic bridging and syncytia formation. To confirm the isolation of a poxvirus, an immunofluorescence test was conducted with human antivaccinia serum (diluted 1:1,000) and goat antihuman immunoglobulin G (IgG) (diluted 1:500, Dako, Roskilde, Denmark). Diffuse cytoplasmic fluorescence confirmed an orthopoxvirus.

Isolates were further characterized by PCR and sequence analysis with primers for the hemagglutinin gene (*9*). Melting curve and sequence analyses confirmed the presence of an orthopoxvirus, most likely CPXV (Figure). Because this PCR assay was designed to differentiate variola virus from other orthopoxviruses but not among nonvariola orthopoxviruses, we developed a CPXV-specific PCR by using nested primer sets within the A-type inclusion protein (ATI) gene. PCR was conducted by using external primers (ATIF1) 5´-GAACTTAATAAGTGTTTCGATA-3´> (forward primer) and (ATIR1) 5´-CAGTAACGTCGGACGATGGAGG-3´ (reverse primer) with nested forward primer ATIF2 5´-GAGGAAGTTAAGAGATTGCGTC-3´ and reverse primer ATIR1. The nucleotide sequences are available from GenBank. Nucleotide sequencing confirmed that the virus isolated from Barbary macaques was a CPXV.

**Figure:**
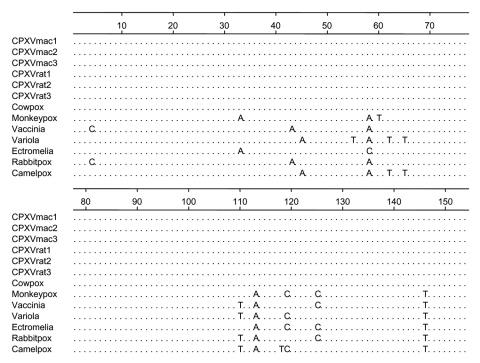
Sequence alignment of the partial hemagglutinin gene of cowpox viruses (CPXV) isolated from Barbary macaques and brown rats. CPXV-2001 strain was isolated from a rat in 2001 from the Netherlands (*5*).

Since all macaques were in the center before disease manifested and they had not been in contact with other animals, other monkeys were tested for CPXV infection by serologic analysis. Serum samples from 16 Barbary macaques (*Macaca sylvanus*), 2 pig-tailed macaques (*M*. *nemestrina*), 2 squirrel monkeys (*Saimiri sciureus*), 2 Japanese macaques (*M*. *fuscata*), 6 cynomolgus macaques (*M*. *fascicularis*), 2 Hamadryas baboons (*Papio hamadryas*), 4 rhesus macaques (*M*. *mulatta*), and 1 vervet (*Cercopithecus aethiops*) were tested with a virus neutralization test (VNT) using a CPXV strain isolated in this study (CPXVmac). Neutralizing titers were determined after 5 days on the basis of complete reduction of a cytopathic effect. At the end of the outbreak, neutralizing serum antibodies were detected in 9 Barbary macaques, 2 pig-tailed macaques, 1 Japanese macaque, 3 cynomolgus macaques, and 1 rhesus monkey. This finding suggested that that all of these animals had been exposed to CXPV. Retrospective serosurveillance showed that only 1 Barbary macaque was seropositive at the start of the outbreak. No swab samples from the animals were available for culture or PCR analysis.

To identify the possible reservoir of the CPXV infection, animals known to be susceptible to CPXV and housed at the sanctuary at the time of the outbreak were tested. These included 4 domestic cats (*Felis catus*), 2 red squirrels (*Sciurus vulgaris*), and 6 prairie dogs (*C*. *ludovicianus*). In addition, 32 wood mice (*A*. *sylvaticus*) and 34 rats (*R*. *norvegicus*) trapped in the area of the sanctuary were tested by serologic analysis and PCR. Only 1 of the tested cats had neutralizing serum antibodies (titer 20) to CPXV. Throat swabs of all 4 cats obtained during the outbreak were negative by both PCRs. These results suggest that cats were not infected by CPXV at the time of the outbreak. Prairie dogs housed at the sanctuary at the time of the outbreak were not infected, as shown by negative PCR results on throat swabs and the absence of orthopoxvirus-specific antibodies.

In contrast, PCR and virus culture showed that 56% of rats tested were infected with CPXV (Table). Sequence comparison of the hemagglutinin and ATI genes of 19 CPXV rat isolates with the Barbary macaque isolates showed identical sequences, which indicated that rats were the most probable source of infection. No significant differences were observed between the genes of CPXVs isolated during this outbreak and previous isolates (Figure).

**Table:** Results for animals tested for cowpox virus infection, the Netherlands, 2003*

Animal tested	VNT	ELISA	Virus isolation	PCR
Barbary macaque (*Macaca sylvanus*)	9/16	9/16	ND	ND
Pig-tailed macaque (*M. nemestrina*)	2/2	2/2	ND	ND
Squirrel monkey (*Saimiri sciureus*)	0/2	0/2	ND	ND
Japanese macaque (*M. fuscata*)	1/2	1/2	ND	ND
Cynomolgus macaque (*M. fascicularis*)	3/6	4/6	ND	ND
Hamadryas baboon (*Papio hamadryas*)	0/2	0/2	ND	ND
Rhesus macaque (*M. mulatta*)	1/4	2/4	ND	ND
Vervet (*Cercopithecus aethiops*)	0/1	0/1	ND	ND
Domestic cat (*Felis catus*)	1/4	3/4	0/4	0/4
Red squirrel (*Sciurus vulgaris*)	0/2	0/2	0/2	0/2
Prairie dog (*Cynomys ludovicianus*)	0/6	0/6	0/6	0/6
Wood mouse (*Apodemus sylvaticus*)	0/32	2/32	0/32	0/32
Brown rat (*Rattus norvegicus*)	ND	11/34	16/28	19/34

*Values are no. positive/no. tested. VNT, virus neutralization test; ELISA, enzyme-linked immunosorbent assay; PCR, polymerase chain reaction, ND, not determined.

Since serum samples collected from trapped dead rats could not be tested by VNT, we developed an indirect enzyme-linked immunosorbent assay (ELISA) based on vaccinia virus, which was validated with sera of monkeys infected with vaccinia virus. Briefly, vaccinia virus was treated with 2% Triton X-100, and 100 ng was added to wells of high-binding plates (Costar, Corning Inc., Corning, NY, USA). Two-fold dilutions of sera starting at 1:20 were added to the plates, and antibodies were detected with horseradish peroxidase–labeled protein A (1:1,000 dilution, Zymed Laboratories, South San Francisco, CA, USA). Titers were expressed as the reciprocal of the highest serum dilution for which an optical density at 450 nm (OD_450_) was >3× the OD_450_ of the negative control. Of 19 rats that tested positive by PCR, only 3 were seropositive, which suggested that CPXV was spreading actively in the rat population. Swabs of all mice tested were negative by both PCRs, and only 2 of 32 mice tested were seropositive by ELISA, indicating that mice were not the source of infection.

## Conclusions

This is the first report describing CPXV infection in captive monkeys. Wild brown rats captured at the sanctuary were infected with CPXV and assumed to be the most probable source of infection. Whether multiple contacts of infected rats with the monkeys contributed to the outbreak or monkey-to-monkey transmission occurred efficiently is not clear. Furthermore, whether monkeys were infected by rats through direct contact or infected excreta is unclear (*10*). Longitudinal field studies are required to clarify if rats could be the principal reservoirs for CPXV. Circulation of CPXV in wild and captive animals, together with decreased immunity against orthopoxviruses in the community, may put animal trappers and handlers at risk for CPXV infection. Our findings show the threat of orthopoxviruses that can cross species barriers, which indicates the importance of developing new vaccines and antiviral drugs against orthopoxvirus infection of humans.
